# Burnout among pediatric residents and junior consultants working at a tertiary care hospital

**DOI:** 10.12669/pjms.35.1.43

**Published:** 2019

**Authors:** Attia Bari, Rizwan Kamran, Farah Haroon, Iqbal Bano

**Affiliations:** 1 Attia Bari, MBBS, D.C.H, M.C.P.S, F.C.P.S., MHPE Associate Professor, Department of Paediatric Medicine, The Children's Hospital and The Institute of Child Health, Lahore, Pakistan; 2 Rizwana Kamran, MBBS, MHPE. Assistant Professor, CMH Lahore Medical College & Institute of Dentistry (NUMS), Lahore, Pakistan; 3 Farah Haroon, MBBS, FCPS (Paediatric Medicine), FCPS (Neonatology) Associate Professor Pediatric Neonatology, Department of Paediatric Medicine, The Children's Hospital and The Institute of Child Health, Lahore, Pakistan; 4 Iqbal Bano, MBBS, F.C.P.S. Associate Professor, Department of Paediatric Medicine, The Children's Hospital and The Institute of Child Health, Lahore, Pakistan

**Keywords:** Burnout, resident, Patient, Healthcare, Questionnaire, Copenhagen Burnout Inventory

## Abstract

**Objective:**

To determine the burnout among postgraduate residents’ and junior consultants in a tertiary care hospital.

**Methods:**

This cross-sectional study was conducted among the postgraduate residents (PGR) and junior consultants (JC) working at The Children’s Hospital Lahore in 2018. Participants were asked to complete *Copenhagen* Burnout Inventory questionnaire about burnout on 5 point Likert scale. ‘100 (always), 75 (often), 50 (sometimes), 25 (seldom) and 0 (never/almost never or according to intensity ranging from ‘a very low degree’ to ‘to a very high degree’. Data was analyzed using SPSS version 22. Three questions were added related to hospital factors but scored separately. Students t-test and chi square test were used to compare the burnout.

**Results:**

A total of 227 participants including 177 PGR and 50 JC completed the questionnaire with a response rate of 84% and 86% respectively. There was a female predominance, 140 participants (61.7%) were female. Majority was from pediatric medicine 173 (76.2%). The mean personal and work related-burnout was high among PGRs as compared to JCs (18.68±5.01vs 16.62±4.57) (p=0.008) and (21.14±5.57 vs. 18.56±5.52) (p=0.004) respectively. Similarly, there was significantly more burnout among pediatric medicine study participants as compared to surgery and diagnostic in all domains (personal BO; p=0.030, work-related BO; p=0.021, patient related BO; 0.033 and hospital related BO; 0.001). No difference were noted based on gender and year of training.

**Conclusion:**

Tertiary care hospital postgraduate residents and junior consultants face moderate burnout. Postgraduate residents had significantly more burnout as compared to junior consultants and majority was from pediatric medicine.

## INTRODUCTION

Burnout is prevalent amongst workers involved in helping professions like teachers, psychologists and physicians.^1^ It is commonly high among healthcare professionals.^2^ Burnout is a state of chronic stress causing various levels of physical and psychological exhaustion suffered by the individual.^3^ In 1981, Maslach and Jackson defined burnout as a three-dimensional syndrome of emotional exhaustion, depersonalization, and lack of personal accomplishment.^4^

Persistent distress and tension among individuals are the root causes of emotional exhaustion and leads to occupational inefficiency, dissatisfaction and frustration. Depersonalization occurs when doctors develop a negative environment and attitude towards their patients or colleagues, leading to the deterioration of the social climate at their workplace. Inefficiency and lack of personal accomplishment is depicted by the inability of person to do his job responsibilities and resulting detachment and untimely retirement.^4^

Several studies revealed a high level of burnout among residents, junior doctors and fellows particularly in their early training phase. Long working duration, demanding jobs and difficult working environments in organizations can cause physical and emotional distress. Moreover, burnout among residents can affect the delivery of safe care, satisfaction of patients and physicians’ emotional well-being.^5^,^6^ Many questionnaires have been developed to measure burnout namely: the Maslach Burnout Inventory,^4^ Copenhagen Burnout Inventory (CBI)^3^ and Oldenburg Burnout Inventory.^7^

Pediatric specialty is a very demanding field and a full-time emergency. In Pakistan, very little literature is published with an outcome of physicians’ stress and job satisfaction particularly of pediatric specialty. We planned this study to measure the level of burnout among pediatric PGRs and JCs of senior registrar (SR) level, and also to find the contributing elements of high burnout. The study assists us to identify and take measures to resolve the issues regarding burnout if it is present and improve quality of life of pediatric residents and ultimately safe patient care.

## METHODS

This study was conducted at The Children’s Hospital and The Institute of Child Health (ICH) Lahore, a largest Pediatric tertiary care hospital in Pakistan. PGRs who joined ICH at least six months prior to the survey and SR/JC working at ICH were included. After taking permission from the hospital ethical committee a voluntary and anonymous questionnaire was distributed to PGRs and JCs.

Burnout was assessed with CBI; a self-administered, pre-tested and pre-validated tool adapted from the one developed by Maslach. The reliability coefficient of the questionnaire is 0.87.^3^ CBI consists of 19-item questionnaire having three subscales: personal burnout (6 items), work burnout (7 items) and patient burnout (6 items) scored on a 5-point Likert scale according to the frequency with which the respondent feels a particular way in relation to his or her work. Twelve items have responses of frequency ranging from; 100 (always), 75 (often), 50 (sometimes), 25 (seldom) and 0 (never/almost never). Seven items use response categories according to intensity ranging from ‘a very low degree’ to ‘to a very high degree’. Item number 10 was negative statement and was reverse coded.

Being a tertiary care referral hospital of a developing country, there are few other factors which may be a cause of burnout among PGRs and JCs so three more questions of associated hospital related factors were added as suggested by hospital ethical committee. These questions were scored separately.

Frequencies and descriptive statistics were examined for each variable. Statistical comparisons were performed between the “burned out” PGR/JC and the “non-burned out” PGR/JC concerning demographic characteristics. Dichotomous variables were compared with the chi-square test. Statistical significance was considered as p < 0.05. In order to look in to each dimension of burnout separately comparisons of each burnout dimension’s mean score for type of specialty was tested by student’s *t*-test.

## RESULTS

In our study 227 participants (177 PGR and 50 JC) from three pediatric subspecialties of medicine, surgery and diagnostic (radiology and pathology) were included. Questionnaire was distributed to 210 PGRs and 58 JC with a response rate of 84% and 86% respectively. There was a female predominance 140 (61.7%) and majority were from pediatric medicine 173 (76.2%). Demographic characteristics for the study participants are shown in the Table-I. The participant’s individual domain burnout scores are shown in Table-II. These scores provide a view of the burnout among the PGRs and JC.

**Table-I:** Characteristics of Residents and Junior Consultants.

Characteristic	Postgraduate Resident (n=177)	Senior Registrar (n=50)
Age	25-30 years	30-40 years
*Gender*		
Males	66 (37.2%)	21 (42%)
Females	111 (62.7%)	29 (58%)
*Specialty*		
Pediatric Medicine	136 (76.9%)	37 (74%)
Pediatric Surgery	21 (11.8%)	06 (12%)
Pediatric Diagnostic	20 (11.3%)	07 (14%)
*Year of postgraduate residency*		
1^st^ Year	64 (36.2%)	
2^nd^ Year	44 (24.9%)	
3^rd^ Year	36 (20.3%)	
4^th^ Year	19 (10.7%)	
Training complete	14 (07.9%)	

**Table-II:** Items and Mean/SD of PGRs and Junior Consultants score in CBI Scale.

Response category of Burnout	Always or To a very high degree (Score 100%)	Often or To a high degree (Score 75%)	Some-times or somewhat (Score 50%)	Seldom or To a low degree (Score 25%)	Never almost never or to a very low degree (Score 0%)

Personal burnout	n (%)	n (%)	n (%)	n (%)	n (%)
How often do you feel tired? ^a^	40 (17.6)	79 (34.8)	67 (29.5)	41 (18.1)	
How often are you physically exhausted? ^a^	31 (13.7)	75 (33)	69 (30.4)	47 (20.7)	05 (2.2)
How often are you emotionally exhausted? ^a^	28 (12.3)	66 (29.1)	67 (29.5)	59 (26)	7 (3.1)
How often do you think: “I can’t take it anymore” ^a^	26 (11.5)	31 (13.7)	60 (26.4)	67 (29.5)	43 (18.9)
How often do you feel worn out? ^a^	14 (6.2)	43 (18.9)	74 (32.6)	78 (34.4)	18 (7.9)
How often do you feel weak and susceptible to illness? ^a^	12 (5.3)	41 (18.1)	64 (28.2)	76 (33.5)	32 (14.1)
*Work-related burnout*				
Do you feel worn out at the end of the working day? ^a^	34 (15)	71 (31.3)	71 (31.3)	40 (17.6)	11 (4.8)
Are you exhausted in the morning at the thought of another day at work? ^a^	30 (13.2)	45 (19.8)	59 (26)	63 (27.8)	30 (13.2)
Do you feel that every working hour is tiring for you? ^a^	14 (6.2)	30 (13.2)	48 (21.1)	87 (38.3)	48 (21.1)
Do you have enough energy for family and friends during leisure time? *(Reverse coding)*	18 (7.9)	59 (26)	67 (29.5)	52(22.9)	31 (13.7)
Is your work emotionally exhausting?^b^	31 (13.7)	58 (25.6)	66 (29.1)	51 (22.5)	21 (9.3)
Does your work frustrate you? ^b^	20 (8.8)	55 (24.2)	44 (19.4)	69 (30.4)	39 (17.2)
Do you feel burnout because of your work? ^b^	22 (9.7)	50 (22)	65 (28.6)	61 (26.9)	29 (12.8)
*Patient-related burnout*				
Do you find it hard to work with patients? ^b^	12 (5.3)	29 (12.8)	57 (25.1)	80 (35.2)	49 (21.6)
Does it drain your energy to work with patients? ^b^	11 (4.80)	43 (18.9)	59 (26)	73 (32.2)	41 (18.1)
Do you find it frustrating to work with patients? ^b^	10 (4.4)	24 (10.6)	52 (22.9)	80 (35.2)	61 (26.9)
Do you feel that you give more than you get back when you work with patients? ^b^	26 (11.5)	44 (19.4)	66 (29.1)	64 (28.2)	27 (11.9)
Are you tired of working with patients?^a^	14 (6.2)	18 (7.9)	50 (22)	85 (37.4)	60 (26.4)
Do you sometimes wonder how long you will be able to continue working with patients?^a^	17 (7.5)	30 (13.2)	50 (22)	76 (33.5)	54 (23.8)
*Hospital/ Logistics-related burnout*				
Do you feel frustrated due to lack of proper laboratory backup?^a^	88 (38.8)	62 (27.3)	41 (18.1)	30 (13.2)	6 (2.6)
Do you feel frustrated due to patient outcome related satisfaction?^b^	32 (14.1)	79 (34.8)	74 (32.6)	36 (15.9)	6 (2.6)
Do you feel frustrated due to huge number of patients which you have to see?^a^	69 (30.4)	56 (24.7)	63 (27.8)	28 (12.3)	11 (4.4)

a Always, often, sometimes, seldom, never/ almost never (scoring: 100%, 75%, 50%, 25%, 0%)

b To a very high degree, to a high degree, somewhat, to a low degree, to a very low degree

The mean personal BO was high among PGRs as compared to JC (p=0.008) and work related-burnout (p=0.004) shown in Table-III. Similarly, there was significantly more burnout among pediatric medicine study participants as compared to surgery and diagnostic in all domains (personal BO; p=0.030, work-related BO; p=0.021, patient-related BO; 0.033 and hospital-related BO; 0.001). There were only three items which showed statistically significant difference between PGR and JC (Table-IV).

**Table-III:** Individual domains burnout score of the study participants.

Burnout Key Domains	Item number	Postgraduate Residents Mean/SD	Junior Consultants Mean/SD	p-value
Personal burnout	1-6	18.68±5.01	16.62±4.57	0.008
Work-related burnout	7-13	21.14±5.57	18.56±5.52	0.004
Client-related burnout	14-19	15.36±5.64	13.86±4.89	0.089
Hospital/ Logistics-related burnout	20-22	11.07±2.70	10.36±2.94	0.108

**Table-IV:** Items with Significant Differences between PGRs and Junior Consultants

Participants	Never almost never or to a very low degree (Score 0%)	Seldom or To a low degree (Score 25%)	Some-times or somewhat (Score 50%)	Often or To a high degree (Score 75%)	Always or To a very high degree (Score 100%)	p-value
***Personal BO: How often do you feel tired?***						
PGR		33 (18.6%)	41 (23.2%)	67 (37.9%)	36 (20.3%)	0.001
Junior Consultant		08 (16%)	26 (52%)	12 (24%)	04 (8%)	
***Personal BO: How often are you physically exhausted?***						
PGR	5 (2.8%)	28 (15.8%)	53 (29.9%)	62 (35%)	29 (16.4%)	0.003
Junior Consultant	0 (0%)	19 (38%)	16 (32%)	13 (26%)	02 (04%)	
***Work related BO: Do you feel worn out at the end of the working day?***						
PGR	9 (5.1%)	27 (15.3%)	48 (27%)	63 (35.6%)	30 (17%)	0.007
Junior Consultant	2 (4%)	13 (26%)	23 (46%)	08 (16%)	04 (8%)	

## DISCUSSION

Globally burnout is generally high among doctors with varying rates by gender, medical specialty and career stage. PGRs are continually challenged to be on the forefront of patient interaction. Intense work demands in residency predispose PGR and high job responsibility on faculty results in burnout. We used CBI and preferred it over the more popular Maslach Burnout Inventory (MBI) because it is available free and had similar properties.

Overall prevalence of a particular condition can be relied upon with a good response rate. Our high response rate is consistent with a longitudinal study published in Ochsner Journal.^8^ A lower response rate 68% and 50% were shown in studies from Spain and UK respectively.^9^,^10^ A study from Pakistan using CBI on medical students showed 35.9% of students confirmed high levels of burnout.^11^ Similarly a review article from China showed overall prevalence of burnout ranging from 66.5 to 87.8% and burnout was higher among doctors working in tertiary care hospitals and who had long working hours of over 40 h/week.^12^ A research from a tertiary care hospital showed contradictory results in which majority had low burn out and 11% were having moderate and only 1.4% had high burnout.^13^ A study from Spain showed that 40% of PGRs had burnout by one year of their residency training.^9^ Our results showed no difference in burnout when different years of training are compared.

In our study, overall personal burnout was quite high as almost 65% of PGR and JC had scores in the range of 50-75%. Our results are consistent with a study from India in which CBI was used and more than half residents showed high scores of personal burnout domain.^14^ Similar results are shown in a research from Saudi Arabia tertiary care hospital in which burnout syndrome was identified in a high proportion (88.5%) of the respondents.^15^ Being the largest tertiary care referral center, the reason of high personal, work-related and patient-related burnout among PGRs is the changing patient profile which PGRs face while their rotation to different wards.

Multiple factors like social or demographic including gender and age can be indirectly responsible for the job-related burnout. We did not find any difference in burnout score based on gender and similar results narrated in a study from Pakistan published in PJMHS that there was no association of gender with the burnout (p-value 0.782).^16^ But conflicting results are shown in another study from Karachi which showed that females doctors had higher burnout scores than male counterparts.^17^ The pediatric medicine residents have more interaction with patients that might be the cause for higher patient-related burnout when compared with diagnostic residents.

### Limitations

Our study is a cross-sectional survey and cannot explain the variation of burnout which can happen over time. Moreover, this is a single centered study limiting the generalization of our results to postgraduate residents and junior consultants of other institutes including both public and private sector.

## CONCLUSION

Burnout is a common problem among postgraduate residents and junior consultants. The high prevalence of burnout demonstrates the need for appropriate strategies to prevent adverse effects on doctors’ quality of life and on the quality of care patients receive.

### Authors Contribution

***AB:*** Main author, conceived idea, manuscript writing.

***RK:*** Literature searching, contributed in manuscript writing, proof reading.

***FH:*** Data collection.

***IB:*** Critical review.
